# Supplementary material for: Beyond words: Relationships between emoji use, attachment style, and emotional intelligence
Source: PLoS One. 2024 Dec 6;19(12):e0308880. doi: 10.1371/journal.pone.0308880 (PMC11623465; doi:10.1371/journal.pone.0308880)
Supplement: Attachment — Submitted filename: Response to the reviewers_Emoji & Attachement.docx [file pone.0308880.s001.docx]

**Response to the Reviewers**

Emily Chenette, PhD
Editor-in-Chief, PLOS ONE
Janvier 27^th^, 2025

Dear Dr. Chenette
We are pleased to resubmit our manuscript re-titled, “Beyond words: Relationships between emoji use, attachment style, and emotional intelligence” for consideration to *PLoS ONE* as an empirical article. We would like to thank the editor and the reviewers for their constructive feedback. We believe that our manuscript is much better now that we have incorporated their suggestions. We are also pleased to hear that the editor and reviewers feel that our paper is novel, relevant, and interesting. In what follows, please find a list of responses to each comment in the order that the comments were received.

**Comments from Reviewer 1**

**Comment 1**

PSYCHOLOGICAL MODELS AND CONCEPTS. Despite being still studied, the Big Five model is often considered limited from a computational point of view, and also criticized from a psychological perspective: the personality traits are considered too heterogeneous and not characteristic; strongly differing with culture; and the related official survey is modeled on a narrow sample with evident psychometric biases, which may reflect in its application to any sample. Such limitations should at least be cited clearly in the paper, in a proper section, if not removing this model from the study.

**Response 1**

Thank you for pointing out this weakness in our paper. Based on this comment, we have made the decision to cut the Big Five from our paper entirely. Our paper now primarily focuses on emoji use, attachment styles, and emotional intelligence, with the latter also explored as a potential moderator. We believe that this change streamlines our article, making it more cohesive and providing a clearer, more focused contribution to the area of study as a whole.

**Comment 2**

On the other hand, attachment theory is an up-to-date, more solid and accepted one, thus more relevant, but weakly introduced with too generalized beliefs, such as "Specifically, warm and responsive child-caregiver relationships facilitate the development of a secure attachment style, whereas inconsistent and unresponsive child-caregiver relationships facilitate the development of insecure attachment styles (Bowlby 1969/1982). Insecure attachment styles are categorized as anxious or avoidant (Brennan et al., 1998)", which stand only a superficial and limiting description, which could lead to misunderstandings and new biases in the perception of non-expert readers. A more in-depth description of the main standing points of the theory is necessary to understand the contribution on the topic.

**Response 2**

This is an excellent point and we appreciate the opportunity to expand upon attachment theory. We have eliminated the too-generalized sentences and added a more robust section to delve into the main points of the theory and the current research associated with attachment styles and the theory as a whole.

**Comment 3**

Also, speaking of "emotional stability" out of the context given in the original research is not correct here, because the term "emotion" conveys a temporary state of the human neurological system, and not a stable state. Thus, the concept should be provided in different terms.

**Response 3**

Thank you. That was indeed a pertinent comment. However, since we removed personality traits from this article, we believe this comment no longer applies to the new manuscript.

**Comment 4**

BIASES. Some information given in the state-of-the-art analysis is evidently including gender bias, such as "in studies of email correspondence from institutional leaders, emojis are associated with positive perceptions of likability and effectiveness for men, but viewed as positive but less appropriate for women (Riordan & Glikson, 2020)", or selection bias, such as "Liu and Sun (2020) found that extraversion and neuroticism (i.e., low emotional stability) were respectively negatively and positively associated with using emojis to avoid awkwardness", which is not useful if given as a knowledge base without highlighting the limits of biases that a strong ethical fundation should avoid to prolong.

A further selection bias to be highlighted could be because "Data were collected in two (2) days, between December 23rd, 2016, and December 25th , 2016", which is a period in which usually a large part of the population lives Christmas (as a religious or family-gathering event), especially in the U.S:, where the study had been carried. Thus, people alone in front of the PC, ready to reply to the survey on Christmas eve and day, may be already related to particular personal or interpersonal situations affecting their feedback.

It is important to refrain that biases present in a study don't make the study bugged, but only limited, and such limitations should be clearly presented to the reader, for a clearer and more precise understanding of results.

**Response 4**

Thank you for your note about biases within the existing literature. We have added the following line to the introduction section to emphasize the presence of these biases: “These studies demonstrate that there may not only be contextual and gender-based differences in frequency and type of use of emojis, but also potential biases towards emoji use (e.g., based on the user’s gender).”

We have also added the following line to the Interpretation section to underline how this consideration relates to our findings on gender differences in emoji use:

“However, although we did not specifically ask participants to consider expectations of emoji use—particularly along gender lines—we acknowledge that women may also feel societal pressure to use emojis in accordance with gendered expectations for communication style, as noted in previous research on these differences (c.f.,, Burgoon & Dillman, 1995; Dovidio et al., 1988; Holmes, 1995; Rogers, 1989; Tannen, 1990). Specifically, Tannen (1990) proposes that women are expected to use more “rapport” talk, which focuses on relationship building as opposed to men who are expected to use more “report” talk, or focus on task-oriented communication.”

With regard to selection bias on our part, we acknowledge this possibility in our Limitations section:

“Based on the data collection window for this study—December 23rd to 26th—the possibility of selection bias should further be noted. Indeed, amongst the U.S.-based population who participated in this study, these dates coincide with a period of religious and/or family-oriented winter holiday celebrations in which individuals may engage in particular interpersonal interactions that influence both their CMC behavior and expectations.”

We further added the following content to the Future Direction section:

“While this study was primarily limited to exploring emoji use across women and men, future research should examine potential differences in larger, more diverse and representative samples based on other dimensions (e.g., gender/sex, sexual orientation, or relationship status). Future research should also extend the data collection window, acknowledge its implication of the recruited samples, or consider examining broader, more longitudinal use of emojis.”

**Comment 5**

METHODS. The choice of Likert scales should be explained, in particular for the 4-items scale, based that an agreement/disagreement Likert scale is generally built on 5 or 7 modes, to avoid assumptions on intermediate states.

**Response 5**

When designing the survey, we elected to present 4-point scales as a method of forcing participants to ‘choose a side’ rather than choosing a neutral scale point. However, we recognize that this limits our generalizability, especially as some research suggests that more expansive scales, and particularly 7-point scales, are most accurate for assessing participants’ opinions and behaviors. We have added the presence of 4-point scales as a limitation: “This study also uses 4-point Likert scales rather than more expansive 7-point scales which are known to provide more accuracy in data collection (cf., Finstad, 2010).”

**Comment 6**

In the introduction, it is stated that the only dimension on which the use of emojis is studied is gender, and in the results gender is again the only demographic data on which results are discussed. Results could be discussed by different subgrouping, whenever even a partial set of classes is sufficiently represented in the data, as it happens already in gender with woman and man being represented, while gender-neutral people are unbalanced.

**Response 6**

We absolutely agree that this topic presents many opportunities for future study along other dimensions that were not highlighted within our study. However, as noted in our Limitations section, the imbalance in participation along these parameters prevented rigorous analysis within this particular research. We have indicated the following in the Limitations section: “In terms of sampling, this study employed an online convenience sample mostly composed of white, educated, married, and English-speaking heterosexual individuals with a limited number of minority-identifying individuals included.” We also added the following line to the Future Directions section: “While this study was primarily limited to exploring emoji use across women and men, future research should examine potential differences in larger, more diverse and representative samples based on other dimensions (e.g., gender/sex, sexual orientation, or relationship status).”

**Comment 7**

CLARITY OF DIFFERENCE BETWEEN CORRELATION AND CAUSATION. Among the main issues in this paper is apparently in the narrative of correlation being "relation", "associations", then -correctly- "correlations", but at the end becoming generalized statements. For instance, a weakly generalized statement is "These results show several associations between emoji use, personality traits, attachment style, and emotional intelligence, albeit with small effects", where the focus of the review is on "effects" which semantics leads more to a cause-effect relations.
Another more generalized statement is "our results further suggest that people’s ability to process emotions may influence virtual communication", where emotions and people's ability to process them largely varies in time, situation, personal and environmental independent variables that are not considered or cannot be caught in this kind of analysis. In general, the meaning of correlation should be more clear, avoiding drawing inferences that overstate its value.

**Response 7**

Thank you very much for this comment. Across the manuscript—and especially in the discussion—we have adjusted our wording to indicate/emphasize correlation not causation. We have inserted additional qualifiers to be more mindful to not overstate the value of the study.

**Comment 8**

REFERENCE LIST. The reference list may be a bit more up-to-date, since the given studies refer to a broad class of elements (emoticons, emojis, stickers) having particular differences, with a narrow application (e.g., positive/negative sentiment), while more recent studies focus more on affective computing and emotion recognition.

**Response 8**

This is absolutely true. We appreciate the attention to detail and have included more recent studies across domains that become the main focus of this manuscript (i.e., attachment theory and emotional intelligence). To elaborate on and render justice to attachment theory and gendered communication patterns, we still elected to keep and add several, more foundational studies for a well-rounded background review.

**Comments from Reviewer 2**

**Comment 1**

I think it will be more interesting to exploit the data a little more because your analysis is a little simple. For instance, analyzing the correlation leaving out one trait (or group of), using features selection techniques to analyze the relevance of some traits related to classify (for instance) the type of emoji used or what EI they have, just to mention some ideas.

**Response 1**

Thank you very much for this relevant comment. Based on it, we decided to explore whether emotional intelligence moderated the link between attachment style and emoji use. While the results were non-significant, the value of null results should not be underestimated.

**Comments from the Editor**

**Comment 1**

Please add in the submission platform the information about the Format for specific study types, Human Subject Research (involving human participants): Indicate the form of consent obtained (written/oral) or the reason that consent was not obtained (e.g. the data were analyzed anonymously)

**Response 1**

Under Method, please note the following line specifying how consent was obtained: “Participants electronically provided informed consent by reading a Study Information Sheet and clicking a box to agree to participate.”

We hope these modifications are to your liking. If there is anything else, please do not hesitate to contact us. Again, we would like to thank the editor and the reviewers for their efficiency and feedback. Your time and help in presenting our paper to the world is extremely appreciated.

Sincerely,

**The Corresponding Author**
