# Supplementary material for: Beyond words: Relationships between emoji use, attachment style, and emotional intelligence
Source: PLoS One. 2024 Dec 6;19(12):e0308880. doi: 10.1371/journal.pone.0308880 (PMC11623465; doi:10.1371/journal.pone.0308880)
Supplement: Attachment — Submitted filename: Response to the reviewers_Emoji & Attachement_RR2.docx [file pone.0308880.s002.docx]

**Response to the Reviewers**

Emily Chenette, PhD
Editor-in-Chief, PLOS ONE
May 6^th^, 2025

Dear Dr. Chenette
We are pleased to resubmit our manuscript re-titled, “Beyond words: Relationships between emoji use, attachment style, and emotional intelligence” for consideration to *PLoS ONE* as an empirical article. We would like to thank the editor and the reviewers for their constructive feedback. We believe that our manuscript is much better now that we have incorporated their suggestions. We are also pleased to hear that the editor and reviewers feel that our paper is novel, relevant, and interesting. In what follows, please find a list of responses to each comment in the order that the comments were received.

**Comments from Reviewer 1**

**Comment 1**: The previous comments have been mostly addressed, except for the reference list: with the new deletions and additions, is now very focused on couple attachment (where attachment is related to a couple in a loving relationship), whereas in the state of the art the term is more related to parental attachment, which is relevant to the following relationships in life, including loving relationship and friendship, as well as any attachment with a leading role.

**Response 1:** Thank you. We have now expanded the introduction to more broadly cover the links between attachment and relationships with family members, co-workers, and friends.

**Comment 2:** This seems a little off-topic. The various papers on loving relationships are not related to "affective computing" as the reviewer asked earlier. Affective computing refers to techniques that use machine learning, data analysis and artificial intelligence for emotion recognition and affective states. A state-of-the-art review, at least an introductory one, should be included.

**Response 2:** Thank you for this comment. We re-integrated in the introduction a more thorough overview about the emoji use literature, which now includes an introductory paragraph about its links with affective computing. Given that this is not the primary topic of this article, we believe that this paragraph should be sufficient and that should readers want to learn more about such a specific

We hope these modifications allows us to present this manuscript to a wider audience.

Sincerely,

**The Corresponding Author**
